# “Prospective Clinical Validation of an Optimized TMD Screening Tool: Results of a Two-Stage Validation Process”

**DOI:** 10.1007/s00784-026-06995-5

**Published:** 2026-07-11

**Authors:** Raphael Matthias Faulhaber, Ingrid Peroz, Simon Peroz

**Affiliations:** 1 Department of Prosthodontics, Geriatric Dentistry and Functional Sciences, Charité Berlin, Berlin, Germany; 2 Department of Prosthodontics, Geriatric Dentistry and Functional Sciences, Charité – Universitätsmedizin Berlin, Berlin, Germany

**Keywords:** Temporomandibular disorder, TMD screening, Validation study, Diagnostic accuracy, Evidence-based diagnostics

## Abstract

**Objectives:**

The aim of this prospective validation study was to evaluate the diagnostic performance of a newly developed TMD screening tool of the German Society for Craniomandibular Function (DGFDT) in its statistically optimized scoring version as well as in a clinically modified version, and to compare both with established screening instruments.

**Materials and methods:**

A total of 121 consecutive patients treated at Charité – Universitätsmedizin Berlin were examined using the DC/TMD as reference standard. The DGFDT TMD screening tool was compared with the “CMD-Kurzbefund” according to Ahlers and Jakstat and the three TMD-related screening questions according to Lövgren et al. In addition to the primary analysis, a predefined sensitivity analysis excluded cases with isolated, painless temporomandibular joint noises (*n* = 110). Diagnostic performance metrics were calculated with 95% confidence intervals using exact binomial methods.

**Results:**

In the primary analysis, the statistically optimized version of the TMD screening tool achieved both sensitivity and specificity of 100.0%, representing the statistical optimum. The clinically modified DGFDT version achieved a sensitivity of 84.6% while maintaining a specificity of 100.0%. In the predefined sensitivity analysis, its sensitivity increased to 98.1%, while specificity remained 100.0%. Established comparator instruments showed lower sensitivities in the primary analysis (“CMD-Kurzbefund”: 73.8%; 3Q/TMD: 75.4%) and in the sensitivity analysis (“CMD-Kurzbefund”: 85.2%; 3Q/TMD: 90.7%).

**Conclusions:**

Both DGFDT screening versions demonstrated superior diagnostic performance in this single-center cohort. The clinically modified version represents a consensus- and evidence-based alternative that reduces the impact of isolated, painless temporomandibular joint noises without relevant loss of diagnostic accuracy.

**Clinical Relevance:**

The DGFDT TMD screening tool provides a standardized and practical instrument for early clinical detection of temporomandibular disorders and may contribute to the standardization of TMD diagnostics.

## Introduction

Temporomandibular disorders (TMD) represent a highly prevalent and clinically complex group of conditions that may cause functional impairment as well as considerable pain and exhibit a pronounced tendency toward chronification.

With regard to diagnostic instruments, the publication of the Research Diagnostic Criteria for Temporomandibular Disorders (RDC/TMD) – and, in particular, their subsequent revision into the Diagnostic Criteria for Temporomandibular Disorders (DC/TMD) – has established an internationally accepted, valid, and standardized diagnostic protocol whose relevance in both clinical practice and research continues to increase. In contrast, despite the large number of published examination forms and screening approaches, no screening instrument has yet achieved sustained implementation in routine clinical practice. Existing instruments include purely anamnestic approaches, such as the TMD Pain Screener, the Fonseca Anamnestic Index (FAI) and the three TMD-related screening questions (3Q/TMD), as well as primarily clinical examination protocols such as the “CMD-Kurzbefund” according to Ahlers and Jakstat. However, systematic reviews have emphasized the heterogeneity and content-related limitations of available instruments, particularly regarding their ability to combine patient-reported symptoms with clinical and functional findings. This is largely attributable to the lack of standardized, reliable, and at the same time practicable screening methods that reliably integrate anamnestic and clinical parameters [1–6]. 

Against this background, the German Society for Craniomandibular Function (DGFDT) developed a TMD screening tool in 2019 that deliberately follows the structural logic of the DC/TMD with the aim of combining relevant anamnestic and clinical findings within a single instrument [7]. In an initial clinical investigation (Validation Study I), this screening tool was evaluated in 120 consecutive patients treated at the Charité dental clinic and compared with established screening procedures, including the “CMD-Kurzbefund” according to Ahlers and Jakstat as well as the three TMD-related screening questions (3Q/TMD) according to Lövgren et al. [1, 2].

The initial statistical analyses demonstrated a marked reduction in the diagnostic performance of the DGFDT TMD screening tool compared with the “CMD-Kurzbefund”, particularly with respect to sensitivity and specificity and, consequently, positive and negative predictive values [1, 2]. A key reason for this finding was identified as the diagnostic decision algorithm of the DC/TMD, especially the high diagnostic weighting of temporomandibular joint noises. Within the DC/TMD framework, temporomandibular joint noises almost invariably lead to a TMD-relevant diagnosis and are also incorporated into the assessment of the “CMD-Kurzbefund”. In contrast, such noises do not indicate the need for further diagnostic procedures in the original DGFDT TMD screening tool (Version A) [1, 7–10].

**Fig. 1:**
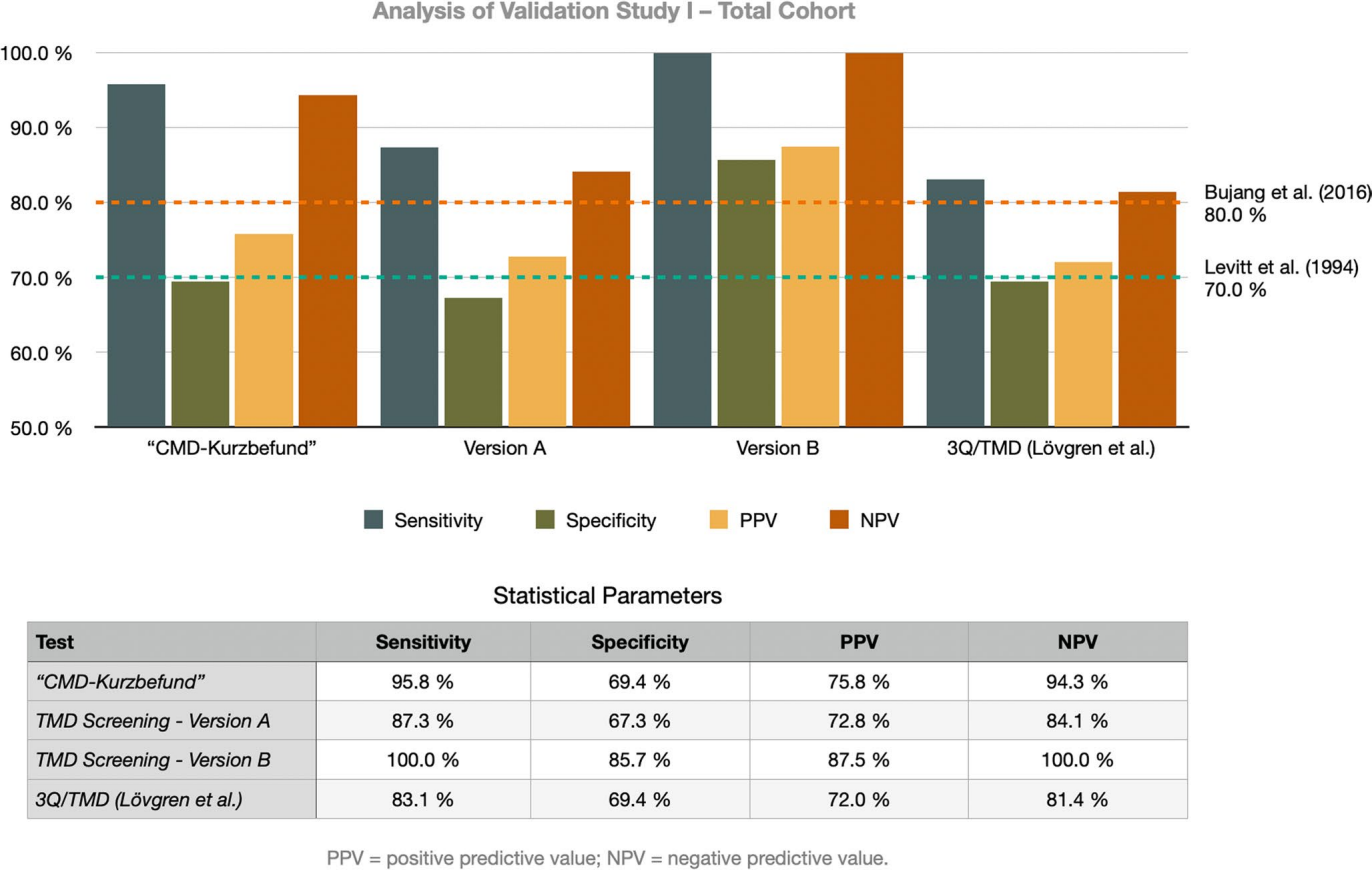
Performance analysis of the DGFDT TMD screening tool in two modifications (Versions A and B) compared with established screening instruments and reference values – total cohort (Validation Study I) [1]

In order to achieve closer alignment with the DC/TMD as the reference standard while simultaneously avoiding the establishment of a purely expert-based screening instrument lacking statistical justification, adaptations of the initial DGFDT TMD screening tool were required. Consequently, in Validation Study I, all individual parameters were systematically evaluated with regard to their correlation with DC/TMD diagnoses. Parameters showing weak or no correlation were removed from the scoring process, differential point values were assigned to the remaining items, and a threshold value was defined within a scoring system above which the screening result was classified as positive and triggered further diagnostic assessment. The resulting statistically optimized scoring version B demonstrated promising diagnostic performance (Fig. 1). However, due to the sample-dependent optimization of the model, its generalizability to the general population was limited [1, 2].

Against this background, the present second validation study (Validation Study II) was initiated to assess the diagnostic performance of the modified screening version derived from Validation Study I under routine clinical conditions. The aim of this study was to re-evaluate sensitivity, specificity, as well as positive and negative predictive values in an independent patient cohort of comparable size, to contextualize the results in relation to established screening instruments, and to assess the suitability of the screening tool for routine clinical use.

For hypothesis formulation, the diagnostic parameters of the “CMD-Kurzbefund” according to Ahlers and Jakstat were used, as in the preceding study, serving as the benchmark to be exceeded (sensitivity = Sens₂ ≤ 92.0% and specificity = Spec₂ ≤ 79.0%). The null hypothesis stated that the DGFDT TMD screening tool would demonstrate both lower sensitivity (Sens₁ < Sens₂) and lower specificity (Spec₁ < Spec₂) compared with the “CMD-Kurzbefund”. Conversely, the alternative hypothesis postulated that the DGFDT TMD screening tool would exceed the “CMD-Kurzbefund” with respect to both sensitivity (Sens₁ > Sens₂) and specificity (Spec₁ > Spec₂) [1, 2, 9, 10]. 

## Materials and methods

Following prior examiner calibration, a total of 121 consecutive adult patients (≥ 18 years) attending the outpatient clinic of the Department of Prosthodontics, Geriatric Dentistry and Functional Sciences at Charité – Universitätsmedizin Berlin were included in this prospective, controlled clinical validation study. Patients were recruited consecutively from routine clinical care, irrespective of sex and independently of the presence of a previously known functional disorder. Thus, the study population comprised both patients presenting with known or suspected TMD-related functional complaints and patients presenting primarily for dental treatment needs. Exclusion criteria were insufficient language proficiency, dementia, and legal guardianship, as these conditions may compromise valid standardized assessment, particularly with regard to the DC/TMD Clinical Examination Protocol, as well as the ability to provide informed consent.

The sample size calculation was based on the benchmark values defined in the hypothesis formulation. For a planned cohort of 120 patients, an expected distribution of 30 patients with TMD and 90 patients without TMD was assumed. Under these assumptions, simulation-based calculations showed that the lower limit of the one-sided 97.5% confidence interval would exceed 69.3% for sensitivity and 65.4% for specificity with a power of 80.0% and a one-sided significance level of α = 0.025. Accordingly, the calculation did not rely on a predefined superiority margin between instruments, but on demonstrating that sensitivity and specificity exceeded these fixed minimum values. Simulations were conducted in R 3.3.2 with 100,000 iterations. The final study cohort comprised 121 consecutive patients.

**Fig. 2:**
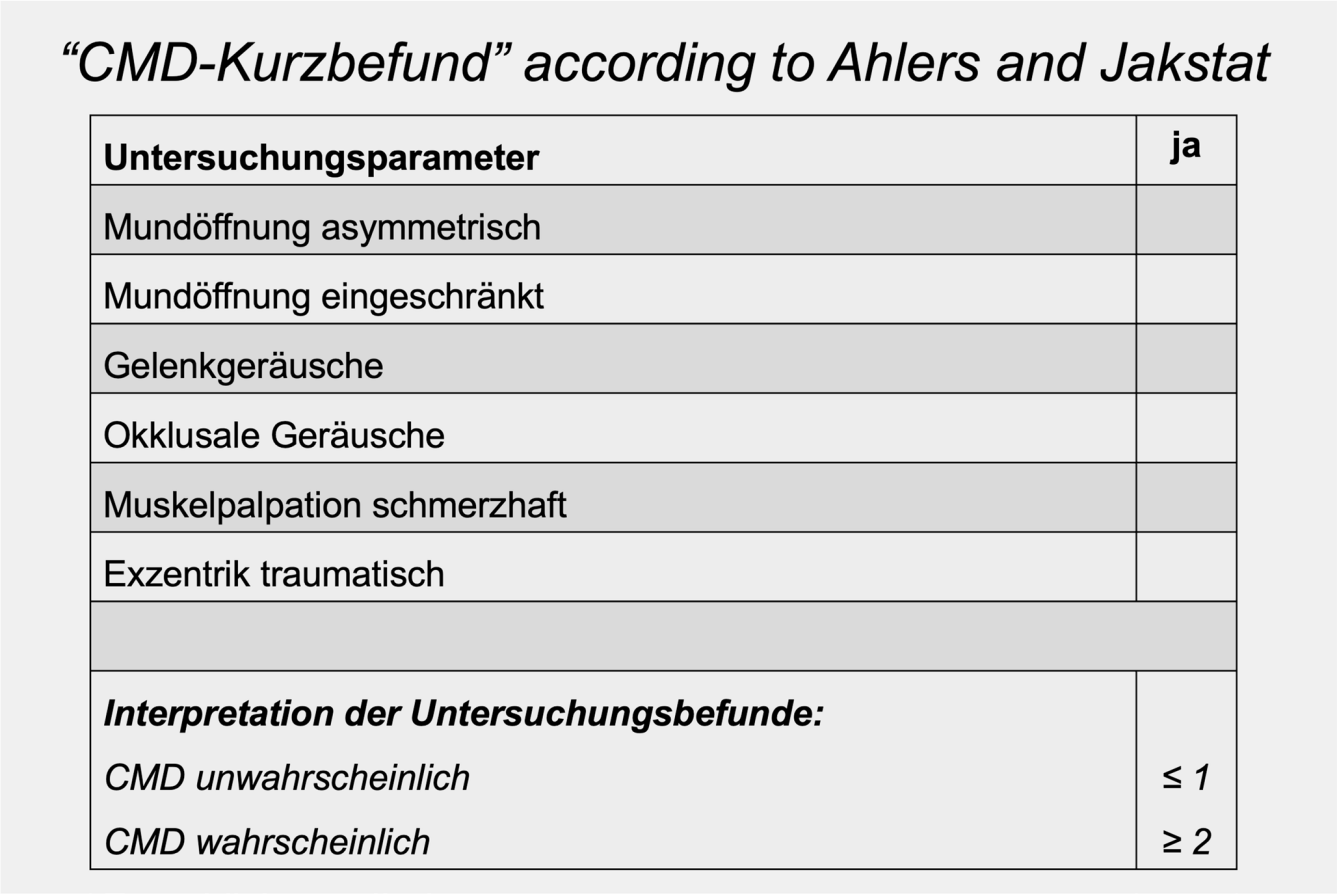
Clinical examination form of the “CMD-Kurzbefund” according to Ahlers and Jakstat [9, 10]. The original German version is shown, as no validated English translation is available. The figure was recreated by the authors for illustration purposes

**Fig. 3:**
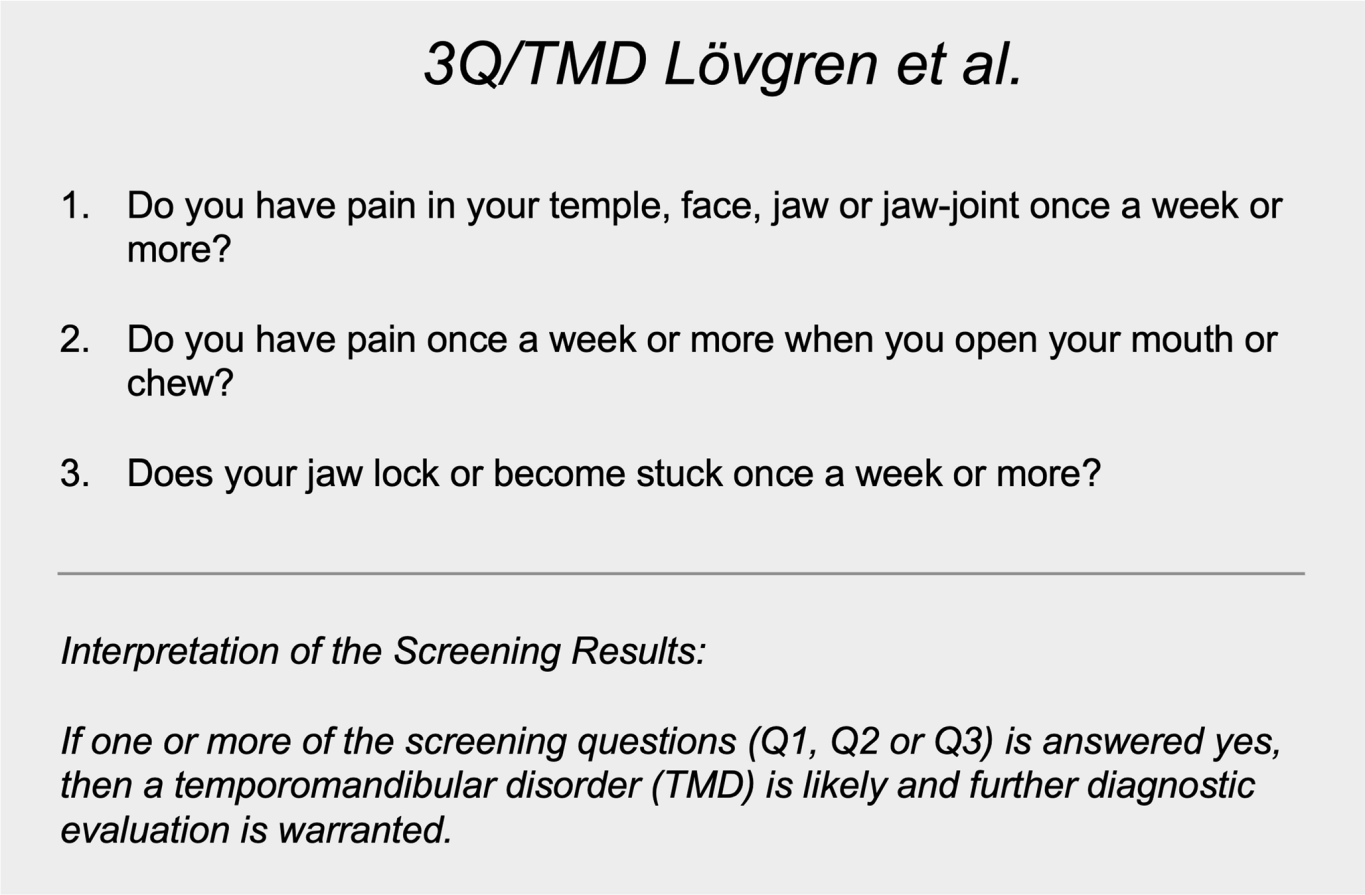
Clinical examination form of the three TMD-related screening questions according to Lövgren et al. [6]

Examinations were performed using the DC/TMD as the reference standard, the “CMD-Kurzbefund” according to Ahlers and Jakstat (Fig. 2), the three TMD-related screening questions according to Lövgren et al. (Fig. 3), and the DGFDT TMD screening tool in its respective modified versions, each assessed with regard to the presence of TMD-relevant diagnoses.

The DC/TMD-based classification followed the published Axis I diagnostic decision algorithms. The standardized assessment included TMD-related history, pain location, pain modification by jaw function, mandibular range of motion, pain on mandibular movement, temporomandibular joint noises, and pain on palpation of the masticatory muscles and temporomandibular joints. Based on these findings, TMD-relevant Axis I diagnoses were assigned and subsequently dichotomized into “TMD present” or “TMD absent” for diagnostic accuracy analysis. A subgroup-specific analysis of individual DC/TMD diagnostic categories was not an aim of the present study and did not affect the calculation of diagnostic performance metrics. However, the distribution of DC/TMD diagnostic subgroups is provided descriptively in Table 1. Axis II psychosocial assessment was not included, as the investigated screening instruments were designed to identify somatic and functional TMD-related findings and to indicate the need for further clinical diagnostic assessment rather than to assess psychosocial impairment [8]. 

**Table 1:** Baseline clinical characteristics and DC/TMD Axis I diagnostic subgroups

DC/TMD Axis I classification	Dental treatment needs (*n* = 66)	Known / suspected TMD (*n* = 55)	Total cohort (*n* = 121)
**No TMD-relevant Axis I diagnosis**	54 (81.8%)	2 (3.6%)	56 (46.3%)
**≥ 1 TMD-relevant Axis I diagnosis**	12 (18.2%)	53 (96.4%)	65 (53.7%)
**Myalgia**	2 (3.0%)	33 (60.0%)	35 (28.9%)
**Arthralgia**	0 (0.0%)	35 (63.6%)	35 (28.9%)
**Disc displacement with reduction**	7 (10.6%)	25 (45.5%)	32 (26.4%)
**Disc displacement with reduction with intermittent locking**	0 (0.0%)	0 (0.0%)	0 (0.0%)
**Disc displacement without reduction with limited opening**	0 (0.0%)	0 (0.0%)	0 (0.0%)
**Disc displacement without reduction without limited opening**	0 (0.0%)	6 (10.9%)	6 (5.0%)
**Degenerative joint disease**	3 (4.5%)	8 (14.5%)	11 (9.1%)

Distribution of recruitment groups and DC/TMD Axis I diagnostic subgroups in the complete study cohort (*n* = 121). Values are presented as n (% within recruitment group). Diagnostic subgroups are not mutually exclusive. Headache attributed to TMD was not listed separately because, according to the DC/TMD diagnostic decision tree, this diagnosis requires a preceding diagnosis of myalgia or arthralgia and was therefore already captured within the binary “TMD present” classification.

Dental treatment needs: patients presenting primarily for dental complaints (*n* = 66).

Known/suspected TMD: patients presenting with known or suspected TMD-related functional complaints (*n* = 55). DC/TMD served as the reference standard

To ensure clear structural differentiation between the examined instruments, the developmental stages of the DGFDT TMD screening tool are referred to using consistent terminology throughout this study. The key differences between the original consensus version, the statistically optimized version, and the clinically modified version are summarized in Table 2.

**Table 2:** Key differences between the DGFDT TMD screening versions

	Version A – original consensus version	Version B – statistically optimized version	Version C – clinically modified version
**Anamnestic pain items**	Included	Included	Included
**Clinical pain / limitation items**	Included	Included	Included
**TMJ noises**	Included; optional diagnostics	High-weight criterion	Reduced weighting
**Occlusal disturbances**	Included; optional diagnostics	Not included	Low-weight criterion
**Traffic-light scoring matrix**	Consensus-based matrix; no point score	Weighted traffic-light scoring matrix	Weighted traffic-light scoring matrix with clinically adapted item weighting
**Cut-off**	No statistically derived cut-off	≥ 3 points	≥ 3 points
**Isolated TMJ noises sufficient for positive screening**	No	Yes	No

Overview of the main structural and scoring-related differences between the original consensus version (Version A), the statistically optimized version (Version B), and the clinically modified version (Version C)

TMJ = temporomandibular joint

Optional diagnostics: extended diagnostic assessment may be considered at the examiner’s discretion but is not mandatory based on this finding in isolation

High-weight criterion: finding sufficient to reach a positive screening result in isolation

Low-weight criterion/reduced weighting: finding contributes to the total score but is not sufficient for a positive screening result in isolation

**Fig. 4:**
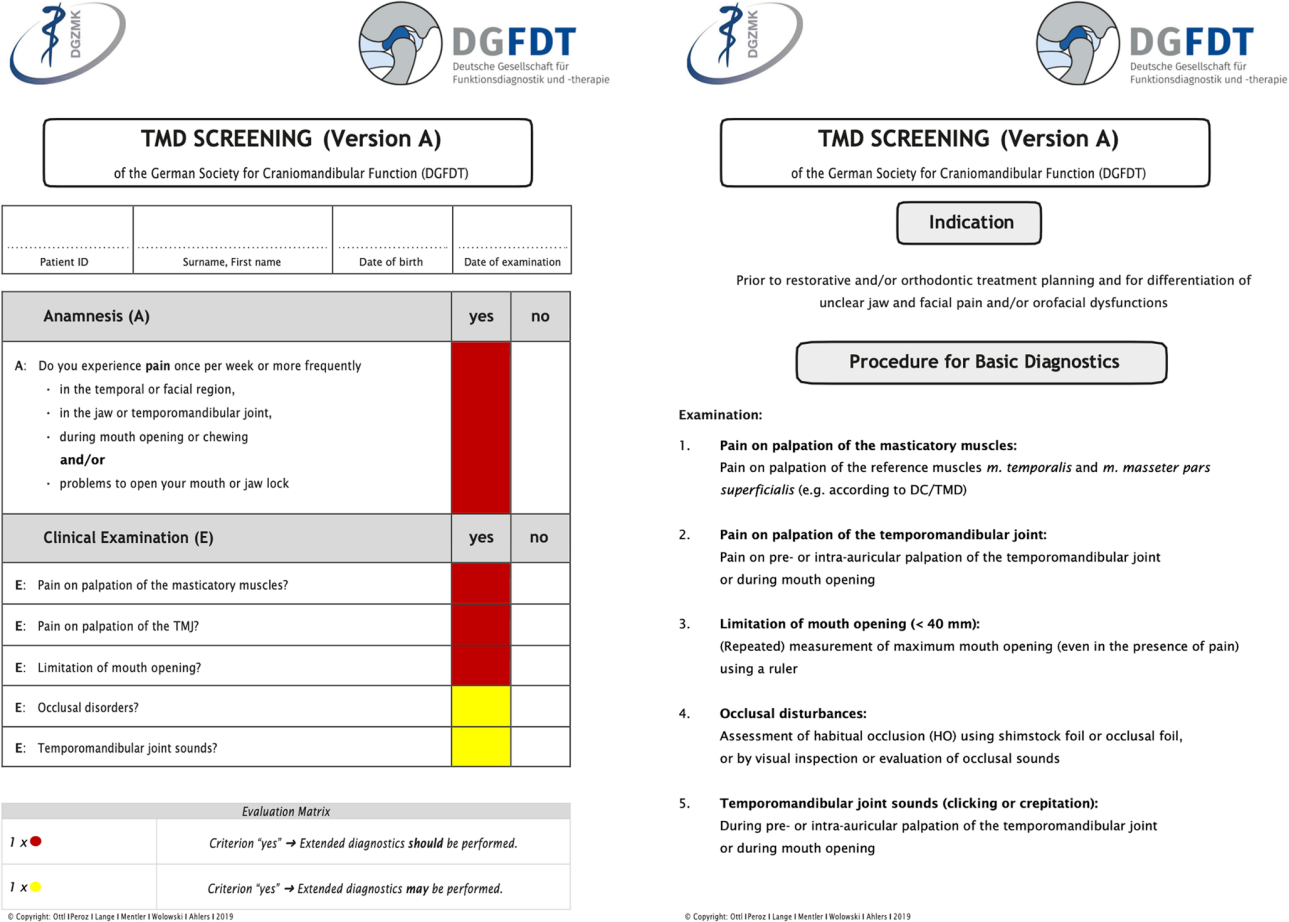
Clinical examination form of the DGFDT TMD screening tool – consensus version (Version A; baseline version prior to statistical optimization) [7]

**Fig. 5:**
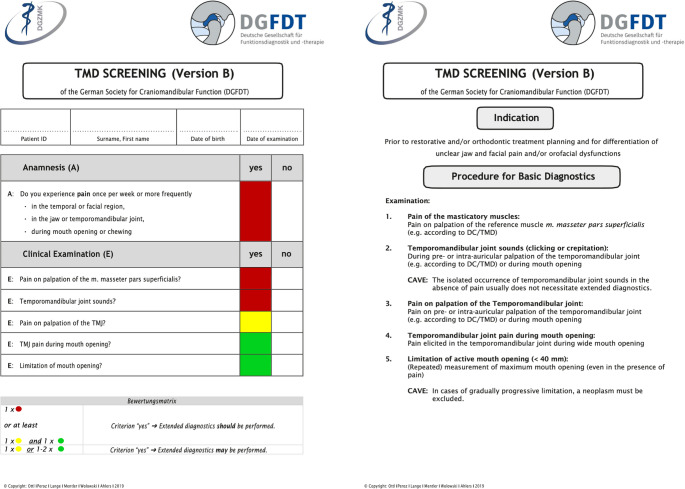
Clinical examination form of the DGFDT TMD screening tool – statistically optimized version (Version B) [1, 7]

The original consensus version developed by the German Society for Craniomandibular Function (Version A; Fig. 4) served as the baseline for subsequent modifications. Based on the results of Validation Study I, as well as on a systematic correlation analysis of all recorded individual parameters combined with receiver operating characteristic (ROC) analysis, the consensus version was optimized (Fig. 5).

**Fig. 6:**
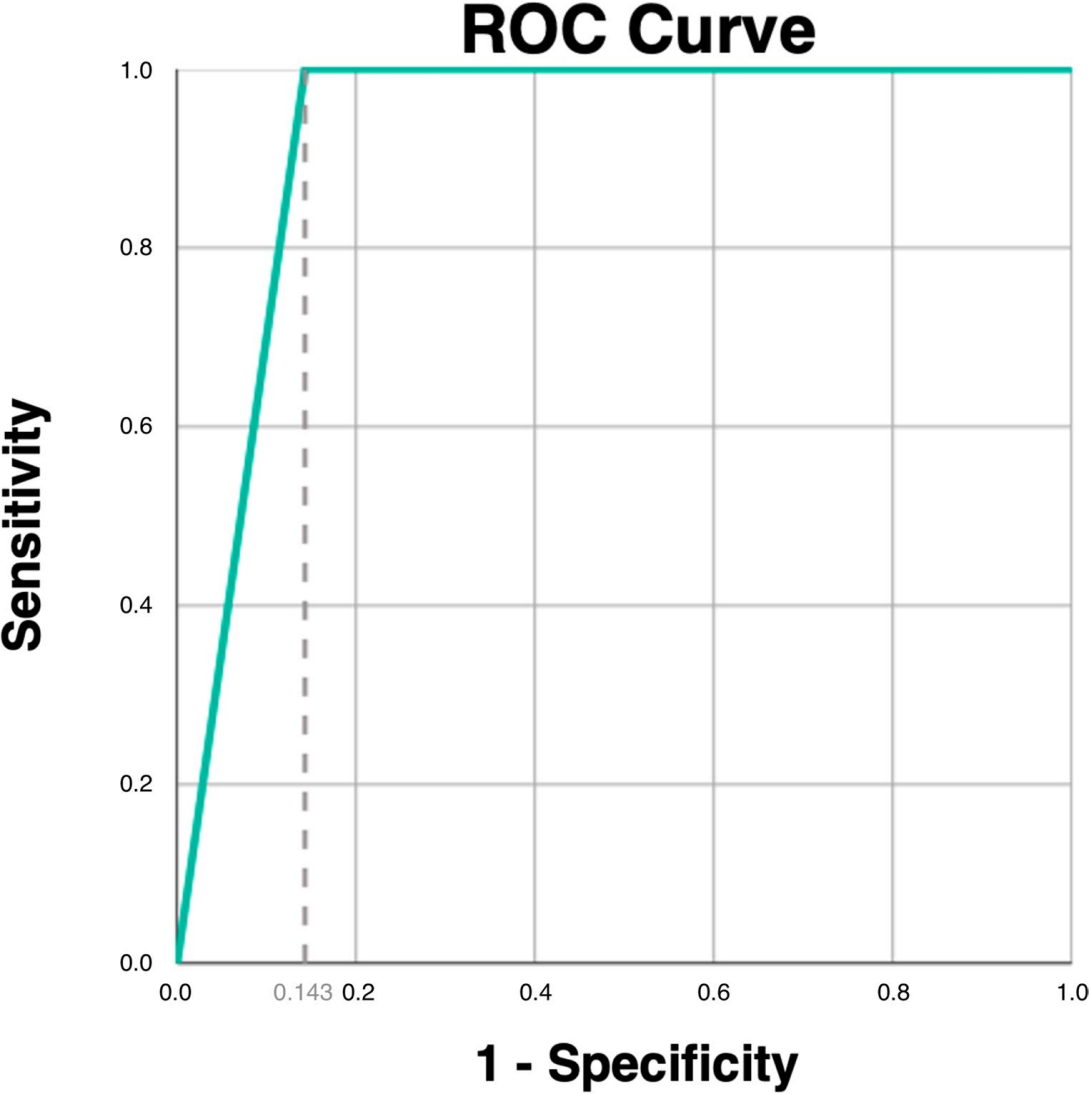
Receiver operating characteristic (ROC) analysis of the developed scoring system (SPSS); x-axis: 1 – specificity, y-axis: sensitivity; illustrated optimum (projection line): sensitivity = 1.0; 1 – specificity = 0.143 [1]

The resulting statistically optimized version of the TMD screening tool (Version B) demonstrated its statistical optimum at a cut-off value of ≥ 3 points, with a sensitivity of 100.0% and a specificity of 85.7% (Fig. 6).

The scoring system illustrated in Fig. 5 corresponds to this version and comprises three anamnestic questions (pain in the temple or facial region, in the jaw or temporomandibular joint, and during mouth opening or chewing occurring at least once per week) and five clinical examination items (palpation of the superficial part of the masseter muscle, temporomandibular joint noises, pain on lateral or dorsal palpation of the temporomandibular joint, pain in the temporomandibular joint during mouth opening, and limitation of mouth opening). The standardized operating instructions and corresponding evaluation matrix allow for structured data acquisition and assessment. The anamnestic questions F1 - F3, pain on palpation of the superficial masseter muscle, and temporomandibular joint noises are defined as “red” criteria, each triggering extended diagnostic assessment even when present in isolation. In addition, the combination of one “yellow” and one “green” criterion - i.e., pain on palpation of the temporomandibular joint and pain in the temporomandibular joint during mouth opening or limitation of mouth opening - also necessitates further diagnostic evaluation, whereas an isolated yellow criterion or one to two green criteria alone do not [1, 2].

**Fig. 7:**
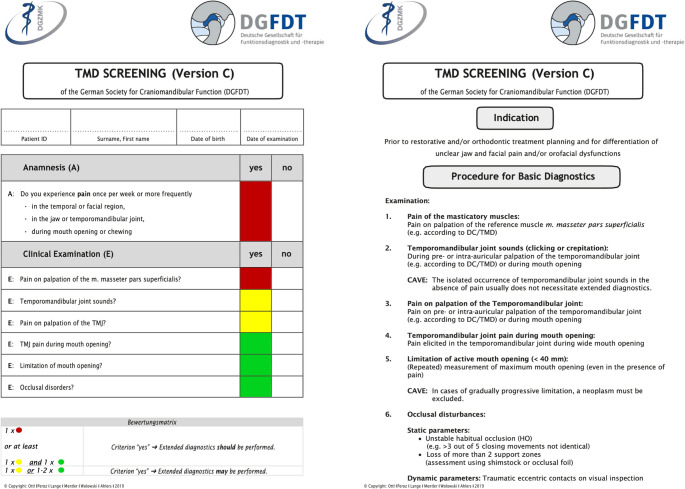
Clinical examination form of the DGFDT TMD screening tool – clinically modified version (Version C) [1, 7]

Independently of the statistical optimization, an additional clinical modification was introduced during further development at the request of the DGFDT. This clinically modified DGFDT version (Version C – Fig. 7) is based on the fundamental structure of the statistically optimized version (Version B) but differs by a reduced weighting of the parameter “temporomandibular joint noises” (classified as a “yellow” criterion) and by the additional inclusion of “occlusal disturbances” as a clinical parameter (classified as a “green” criterion). Occlusal disturbances were subdivided into static and dynamic components. Static criteria comprised unstable habitual occlusion, defined as a non-identical intercuspal position in more than three out of five repetitions during repeated mandibular opening and closing, as well as the loss of more than two posterior support zones, assessed using Shimstock foil or occlusal indicator film. Dynamic occlusal disturbance was defined as traumatic eccentric contacts identified by visual inspection, typically indicated by loss of canine guidance due to attrition, in accordance with the criteria of the “CMD-Kurzbefund” described by Ahlers and Jakstat [1, 9].

The aim of these modifications was to enable a more differentiated clinical assessment, particularly with respect to avoiding potential overdiagnosis in cases of isolated, painless temporomandibular joint noises. The inclusion of occlusal disturbances was not primarily driven by diagnostic necessity but rather by the intent to provide a differentiated clinical documentation of baseline findings. Especially prior to extensive prosthetic or orthodontic treatment, the documentation of occlusal abnormalities is of considerable forensic relevance, as it serves to document pre-existing conditions and allows for transparent reconstruction of baseline clinical findings [1, 2].

Despite these adjustments, the clinically modified Version C demonstrated diagnostic performance values in Validation Study I that were close to the previously determined statistical optimum (sensitivity 84.5%, specificity 93.9%) [1, 2]. Both Version B and Version C were prospectively evaluated in the present study.

All examinations were performed by one calibrated examiner in a standardized clinical sequence. First, the screening instruments under comparison were completed, including the “CMD-Kurzbefund”, the 3Q/TMD, and the DGFDT TMD screening tool. The examiner was not blinded to the screening results, as all instruments were administered within the same clinical session and the scoring algorithms of the screening tools were directly apparent from the recorded findings. Thus, no examiner blinding was implemented. To reduce subjective interpretation, all instruments were applied according to predefined scoring rules and standardized documentation forms.

The resulting anamnestic and clinical data were pseudonymized, digitally recorded, and evaluated using standardized scoring matrices. For statistical analysis, all TMD-relevant diagnoses were combined into a binary outcome variable (“TMD present” = 1; “TMD absent” = 0) and analyzed using IBM SPSS Statistics, version 27.0 (Mac). Diagnostic validity of the investigated instruments was assessed using contingency tables and chi-square testing. Sensitivity, specificity, positive predictive value, and negative predictive value, each with corresponding 95% confidence intervals, were calculated using 2 × 2 contingency tables (Tables 3 and 4), with the DC/TMD serving as the reference standard.

**Table 3:** 2 × 2 contingency tables – total study cohort (n = 121)

Test	TP	FP	FN	TN
**“CMD-Kurzbefund”** (Ahlers and Jakstat)	48	14	17	42
**TMD Screening - Version B** (DGFDT)	65	0	0	56
**TMD Screening - Version C** (DGFDT)	55	0	10	56
**3Q/TMD** (Lövgren et al.)	49	0	16	56

TP = true positive (screening positive, TMD present according to DC/TMD)

FP = false positive (screening positive, no TMD according to DC/TMD)

FN = false negative (screening negative, TMD present according to DC/TMD)

TN = true negative (screening negative, no TMD according to DC/TMD)

DC/TMD served as the reference standard

Given the discrepancy identified in Validation Study I between the DC/TMD diagnostic decision algorithm and the assessment of isolated, painless temporomandibular joint noises in the investigated screening instruments, a predefined sensitivity analysis was conducted in addition to the primary analysis. This analysis was based on the high prevalence of such findings in the general population (approximately 30%) [11, 12], their low tendency toward aggravation [13, 14], and current guideline-based recommendations indicating that isolated, painless temporomandibular joint noises do not justify therapeutic intervention in the absence of pain or functional impairment [15, 16]. Following individual case review, patients were excluded from the sensitivity analysis if temporomandibular joint noise was the only TMD-relevant finding, without TMD-related pain in the history and without pain on mandibular movement or palpation. Patients with any pain-related TMD finding, limitation of mouth opening, or additional clinically relevant TMD diagnosis remained included. Subsequently, diagnostic performance metrics of all investigated screening instruments were recalculated to systematically assess the influence of this parameter on sensitivity, specificity, and predictive values. Confidence intervals were determined using exact binomial calculation according to the Clopper–Pearson method.

**Table 4:** 2 × 2 contingency tables – cohort after exclusion of isolated, painless temporomandibular joint noises (*n* = 110)

Test	TP	FP	FN	TN
**“CMD-Kurzbefund”** (Ahlers and Jakstat)	46	14	8	42
**TMD Screening - Version B** (DGFDT)	54	0	0	56
**TMD Screening - Version C** (DGFDT)	53	0	1	56
**3Q/TMD** (Lövgren et al.)	49	0	5	56

TP = true positive (screening positive, TMD present according to DC/TMD)

FP = false positive (screening positive, no TMD according to DC/TMD)

FN = false negative (screening negative, TMD present according to DC/TMD)

TN = true negative (screening negative, no TMD according to DC/TMD)

DC/TMD served as the reference standard

**Table 5:** Diagnostic performance metrics with 95% confidence intervals (CI)

	Test	Sensitivity (95% CI)	Specificity (95% CI)	PPV (95% CI)	NPV (95% CI)
Total	“**CMD-Kurzbefund**” (Ahlers/ Jakstat)	73.8 (61.5–84.0)	75.0 (61.6–85.6)	77.4 (65.0–87.1)	71.2 (57.9–82.2)
Total	***TMD Screening - Version B*** *(DGFDT)*	100.0 (94.5–100.0)	100.0 (93.6–100.0)	100.0 (94.5–100.0)	100.0 (93.6–100.0)
Total	***TMD Screening - Version C*** *(DGFDT)*	84.6 (73.5–92.4)	100.0 (93.6–100.0)	100.0 (93.5–100.0)	84.8 (73.9–92.5)
Total	***3Q/TMD*** *(Lövgren et al.)*	75.4 (63.1–85.2)	100.0 (93.6–100.0)	100.0 (92.7–100.0)	77.8 (66.4–86.7)
Excluded	***“CMD-Kurzbefund”*** *(Ahlers/ Jakstat)*	85.2 (72.9–93.4)	75.0 (61.6–85.6)	76.7 (64.0–86.6)	84.0 (70.9–92.8)
Excluded	***TMD Screening - Version B*** *(DGFDT)*	100.0 (93.4–100.0)	100.0 (93.6–100.0)	100.0 (93.4–100.0)	100.0 (93.6–100.0)
Excluded	***TMD Screening - Version C*** *(DGFDT)*	98.1 (90.1–100.0)	100.0 (93.6–100.0)	100.0 (93.3–100.0)	98.2 (90.6–100.0)
Excluded	***3Q/TMD*** *(Lövgren et al.)*	90.7 (79.7–96.9)	100.0 (93.6–100.0)	100.0 (92.7–100.0)	91.8 (81.9–97.3)

Sensitivity, specificity, positive predictive value (PPV), and negative predictive value (NPV) with corresponding 95% confidence intervals (Clopper–Pearson)

Total: complete study cohort (*n* = 121)

Excluded: cohort after exclusion of isolated, painless temporomandibular joint noises (sensitivity analysis; *n* = 110)

DC/TMD served as the reference standard

The study was conducted in accordance with the ethical principles of medical research as outlined in the Declaration of Helsinki. Data acquisition and analysis were performed following approval by the Ethics Committee of Charité-Universitätsmedizin Berlin (ethics approval number EA1/251/21).

## Results

**Fig. 8:**
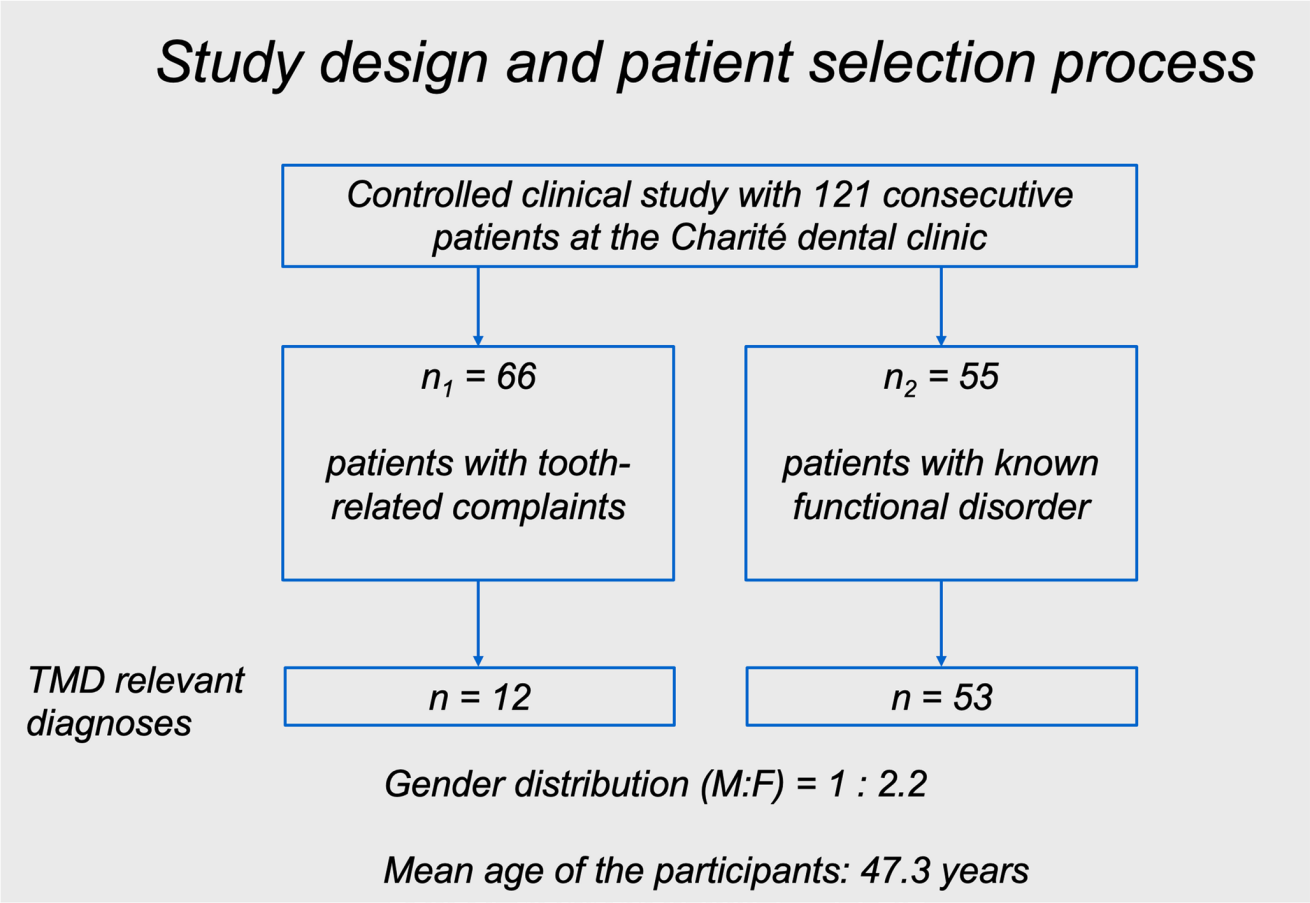
Descriptive data and study design

Of the 121 consecutive patients examined at the Charité dental clinic, 55 patients presented with a function-related disorder already known from medical history, whereas 66 patients attended the clinic primarily due to dental complaints. Within the group of patients with a previously known functional disorder, 53 of 55 patients were assigned a TMD-relevant diagnosis according to the DC/TMD criteria, while such a diagnosis was identified in 12 of 66 patients in the comparison group. The male-to-female ratio was 1:2.2, with a mean age of the study population of 47.3 years (Fig. 8) [1, 2].

**Fig. 9:**
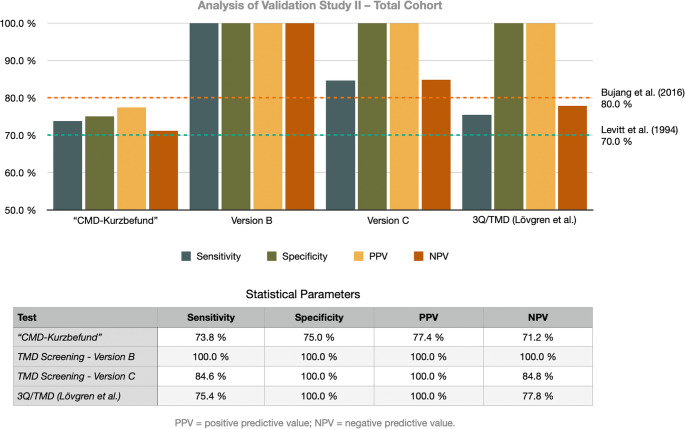
Performance analysis of the DGFDT TMD screening tool in two modifications (Versions B and C) compared with established screening instruments and reference values – total cohort (Validation Study II)

In the statistical analysis based on contingency tables (Fig. 9), the “CMD-Kurzbefund” achieved a sensitivity of 73.8% and a specificity of 75.0%. Both values exceeded the threshold of 70.0% proposed by Levitt et al., above which a screening instrument may be considered valid [17].

The statistically optimized version of the DGFDT TMD screening tool (Version B) confirmed the diagnostic performance values predicted in Validation Study I. With a sensitivity of 100.0% and a specificity of 100.0%, it represented the statistical optimum. By exceeding the 80% threshold for both parameters, Version B also fulfilled more stringent diagnostic accuracy thresholds proposed in the literature [18, 19].

The clinically modified DGFDT version (Version C) achieved a sensitivity of 84.6% and a specificity of 100.0%. As expected, these values were lower than those of the statistically optimized version but nevertheless exceeded the threshold values commonly applied to assess diagnostic validity.

The three TMD-related screening questions according to Lövgren et al. demonstrated a specificity of 100.0% in the present study, representing a marked improvement compared with Validation Study I (previously 69.4%). Sensitivity remained lower at 75.4% and thus below the values observed for both DGFDT screening versions [1].

In light of these findings and considering the particular role of the DC/TMD as the reference standard, further statistical analyses were conducted. Within the DC/TMD diagnostic decision algorithm, isolated temporomandibular joint noises without accompanying pain symptoms regularly result in a TMD-relevant diagnosis, which may substantially influence diagnostic performance metrics, particularly in screening studies.

As part of the predefined sensitivity analysis, eleven patients presenting exclusively with isolated, painless temporomandibular joint noises were excluded following individual case review. As a result, the sample size was reduced to 110 participants, comprising 51 patients with a previously known functional disorder and 59 patients presenting primarily with dental treatment needs. According to the DC/TMD reference standard, 54 patients were classified as TMD-positive and 56 as TMD-negative in this cohort.

**Fig. 10:**
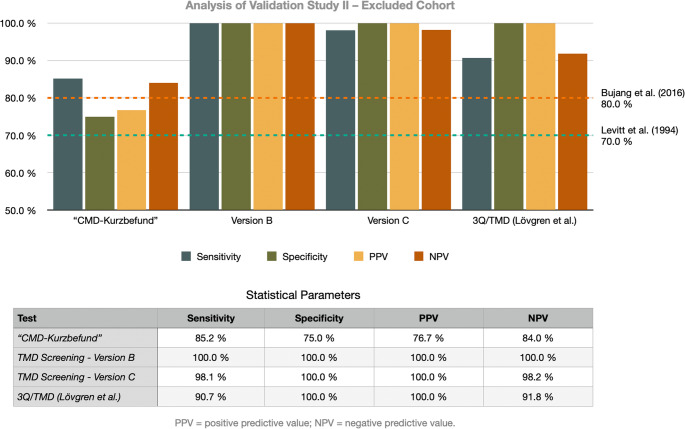
Performance analysis of the DGFDT TMD screening tool in two modifications (Versions B and C) compared with established screening instruments and reference values – excluded cohort (Validation Study II)

Reanalysis of the data following exclusion of these cases (Fig. 10) again confirmed the statistical optimum of the statistically optimized DGFDT TMD screening version (Version B), with a sensitivity of 100.0% and a specificity of 100.0%. Diagnostic performance of the remaining screening instruments also improved under these conditions: the “CMD-Kurzbefund” achieved a sensitivity of 85.2%, and the three TMD-related screening questions according to Lövgren et al. reached a sensitivity of 90.7%. The clinically modified DGFDT version C also showed an increase in sensitivity to 98.1%, while specificity remained unchanged across all instruments. With this result, the clinically modified DGFDT version was only 1.9 percentage points below the statistical optimum and represented the statistically closest alternative.

The calculated 95% confidence intervals (Table 5) confirmed the high precision of the estimated diagnostic performance metrics.

## Discussion

The results of the present second validation study confirm the diagnostic performance parameters calculated in the first validation study for the DGFDT TMD screening tool modified in the form of a scoring system, which had not previously been prospectively evaluated in an independent patient cohort [1]. However, as both validation studies were conducted at the same university-based center and by the same examiner, the present investigation should be interpreted as a prospective single-center validation rather than as a true external multicenter validation. The consistently very high sensitivity and specificity support the methodological soundness and clinical viability of the implemented modifications – particularly the differentiated weighting of individual parameters and the definition of a clear cut-off value.

The perfect diagnostic performance observed for the statistically optimized Version B should nevertheless be interpreted cautiously. Since this version was derived from sample-dependent statistical optimization in Validation Study I and was subsequently re-evaluated within the same institutional setting, residual overfitting and center-specific effects cannot be fully excluded. External validation in independent, multicenter, and more heterogeneous populations is therefore required to confirm the generalizability of these promising findings.

In comparison with established screening instruments such as the “CMD-Kurzbefund” according to Ahlers and Jakstat and the three TMD-related screening questions according to Lövgren et al., both modified DGFDT screening versions showed higher diagnostic performance in the present cohort. Their sensitivity and specificity exceeded the 70% threshold proposed by Levitt et al. for screening instruments and, to a large extent, also exceeded the more stringent 80% threshold discussed in the literature [17–19]. Nevertheless, given the single-center design, the selected referral-based study population, and the absence of external multicenter validation, the instrument should currently be interpreted as a screening tool for identifying patients who may require further structured TMD-related diagnostic assessment, rather than as a standalone diagnostic instrument.

As discussed in the preceding study, the DC/TMD used as the reference standard is associated with known limitations, particularly with regard to the high diagnostic weighting of temporomandibular joint noises, which within the diagnostic decision algorithm may lead to a TMD-relevant diagnosis even in the absence of concomitant pain symptoms [1, 8]. Despite these limitations, the DC/TMD was retained as the reference standard due to the lack of internationally established alternatives.

In light of this well-recognized issue, a predefined sensitivity analysis was performed in addition to the primary analysis, excluding patients presenting with isolated, painless temporomandibular joint noises. This additional analysis resulted in a more realistic assessment of this parameter and was associated with marked changes in the performance metrics of all investigated screening instruments, particularly with improved sensitivity while specificity remained unchanged or high. In this context, the clinically modified DGFDT version (Version C) demonstrated only a reduction in sensitivity of 1.9 percentage points compared with the statistical optimum, while specificity was fully preserved. This enables a more differentiated clinical assessment without increasing the risk of systematic over- or underdiagnosis.

The statistical stability of these findings is further supported by the calculated 95% confidence intervals (Table 5), particularly for Version B and for the specificity estimates of Version C. However, the width of some intervals, especially for sensitivity estimates in the primary analysis, reflects the limited sample size and should be considered when interpreting the results.

The study population was recruited from a supraregional university-based healthcare facility, which is disproportionately frequented by patients with pre-existing TMD-related diagnoses or symptoms. This results in a selection bias that limits the generalizability of the observed prevalence data to the general population. At the same time, the high proportion of TMD-positive cases must be considered when interpreting predictive values, as these parameters are strongly influenced by disease prevalence. In a general dental practice setting, where the prevalence of clinically relevant TMD is expected to be considerably lower, the positive predictive value of a screening instrument would likely decrease, whereas the negative predictive value would likely increase. Accordingly, the present results primarily support the use of the DGFDT TMD screening tool as a structured instrument for identifying patients who may require further functional diagnostic assessment, while its performance in lower-prevalence primary care populations remains to be confirmed in future studies [1, 20]. Independent of the described selection bias, the predisposition of the female sex – especially at more advanced age – was once again confirmed [1, 21–24].

Furthermore, the presence of information bias cannot be excluded, as a proportion of patients were referred, meaning that symptoms were already known or that a suspected or established TMD diagnosis had been made externally. Additional methodological limitations arise from the single-examiner design and the absence of examiner blinding. Since the screening instruments were completed before DC/TMD-based diagnostic classification and their scoring algorithms were directly apparent to the examiner, observer bias cannot be excluded. Moreover, the single-examiner design precluded assessment of interrater reliability. These aspects should be considered when interpreting the high diagnostic performance observed in the present cohort.

Overall, however, one of the central limitations of the first validation study – namely the problematic assessment of isolated temporomandibular joint noises – was successfully addressed within the framework of the further developed screening concept. The DGFDT TMD screening tool thus represents a structured screening approach aligned with national and international diagnostic concepts and supported by both evidence-based optimization and expert consensus.

## Conclusion

The DGFDT TMD screening tool modified in the form of a scoring system clearly confirmed its diagnostic performance in the present validation study. In the primary analysis, the statistically optimized version B achieved both sensitivity and specificity of 100%, thereby once again confirming the previously identified statistical optimum. This high diagnostic accuracy was fully preserved in an additional sensitivity analysis that accounted for the known limitations of the DC/TMD as the reference standard and clearly exceeded the performance of established screening instruments.

The clinically modified version C requested by the German Society for Craniomandibular Function – characterized by a reduced weighting of the parameter “temporomandibular joint noises” and the additional inclusion of the parameter “occlusal disturbances” – also demonstrated excellent diagnostic performance. In the primary analysis, Version C achieved a sensitivity of 84.6% and a specificity of 100.0%. In the predefined sensitivity analysis excluding isolated, painless temporomandibular joint noises, sensitivity increased to 98.1%, while specificity remained 100.0%. Both versions therefore showed superior diagnostic performance compared with established screening procedures in the present cohort. The calculation of 95% confidence intervals further supported the robustness of the results, although their interpretation should consider the limited sample size and single-center design.

In the final interpretation of the study findings, preference was deliberately given to the clinically modified DGFDT version, despite its marginally lower statistical performance compared with the optimum. This decision was driven by clinical relevance: the differentiated assessment of temporomandibular joint noises substantially reduced the risk of systematic overdiagnosis and subsequent overtreatment. The screening tool therefore represents not only a statistically valid but also a clinically balanced instrument.

A screening tool must not only demonstrate high sensitivity and specificity but also be practicable, reliable, and economically reasonable. The presented DGFDT TMD screening tool meets these criteria: it is clearly structured, easily integrated into routine clinical practice, and contributes substantially to improved and standardized care of patients with temporomandibular disorders.

Following completion of both the primary clinical investigation (Validation Study I) and the present prospective validation study (Validation Study II), a consensus- and evidence-based screening instrument is now available that is aligned with national and international standards, methodologically robustly validated, and allows for differentiated clinical application. As such, it makes a substantial contribution to the urgently needed standardization of TMD diagnostics.

From a future perspective, the DGFDT TMD screening tool should therefore be understood as a structured entry point into a stepwise diagnostic process rather than as a standalone diagnostic instrument. In this role, it may contribute to bridging the gap between internationally standardized TMD diagnostics and the need for practicable screening procedures in routine clinical care. Further external validation in independent, multicenter, and more heterogeneous populations is required to confirm the generalizability, reproducibility, and clinical impact of the present findings.

## Data Availability

The datasets generated and analysed during the current study are available from the corresponding author on reasonable request. Due to privacy and ethical restrictions, the data are not publicly available.
